# Trypsin-like Inhibitor Domain (TIL)-Harboring Protein Is Essential for *Aedes aegypti* Reproduction

**DOI:** 10.3390/ijms23147736

**Published:** 2022-07-13

**Authors:** Chinmay Vijay Tikhe, Victor Cardoso-Jaime, Shengzhang Dong, Natalie Rutkowski, George Dimopoulos

**Affiliations:** 1 W. Harry Feinstone Department of Molecular Microbiology and Immunology, Johns Hopkins Bloomberg School of Public Health, Baltimore, MD 21205, USA; ctikhe1@jhu.edu (C.V.T.); vcardos1@jhu.edu (V.C.-J.); sdong13@jhu.edu (S.D.); nrutkow1@jhmi.edu (N.R.); 2 Johns Hopkins Malaria Research Institute, Johns Hopkins Bloomberg School of Public Health, Baltimore, MD 21205, USA

**Keywords:** *Aedes aegypti*, reproduction, trypsin inhibitor, mosquitoes, CRISPR-Cas9, dengue

## Abstract

Cysteine-rich trypsin inhibitor-like domain (TIL)-harboring proteins are broadly distributed in nature but remain understudied in vector mosquitoes. Here we have explored the biology of a TIL domain-containing protein of the arbovirus vector *Aedes aegypti*, cysteine-rich venom protein 379 (CRVP379). CRVP379 was previously shown to be essential for dengue virus infection in *Ae. aegypti* mosquitoes. Gene expression analysis showed CRVP379 to be highly expressed in pupal stages, male testes, and female ovaries. CRVP379 expression is also increased in the ovaries at 48 h post-blood feeding. We used CRISPR-Cas9 genome editing to generate two mutant lines of CRVP379 with mutations inside or outside the TIL domain. Female mosquitoes from both mutant lines showed severe defects in their reproductive capability; mutant females also showed differences in their follicular cell morphology. However, the CRVP379 line with a mutation outside the TIL domain did not affect male reproductive performance, suggesting that some CRVP379 residues may have sexually dimorphic functions. In contrast to previous reports, we did not observe a noticeable difference in dengue virus infection between the wild-type and any of the mutant lines. The importance of CRVP379 in *Ae. aegypti* reproductive biology makes it an interesting candidate for the development of *Ae. aegypti* population control methods.

## 1. Introduction

The anthropophilic yellow fever mosquito *Aedes aegypti* is considered as the principal vector of multiple arboviruses, being responsible for the transmission of yellow fever virus (YFV), dengue virus (DENV), Zika virus (ZIKV), Chikungunya virus (CHIKV), and a newly emerging pathogen, Mayaro virus (MAYV). Dengue fever is the most widespread mosquito-borne viral disease, reported in at least 130 countries. In the last 20 years, the prevalence of dengue fever has increased eightfold. In 2019 alone, approximately 5.2 million cases of dengue were reported globally [1]. Cases are mainly reported from tropical and subtropical countries in Asia and South America. The staggering number of dengue fever cases adds a significant burden to the medical and economic infrastructure of many developing countries [2,3]. The lack of effective medical interventions such as vaccines and drugs make the control of dengue fever challenging [4,5].

Mosquito control with chemical insecticides remains the primary method for controlling *Ae. aegypti* mosquitoes. However, in recent years, multiple mechanisms of insecticide resistance against a majority of insecticides have been reported in *Ae. aegypti* mosquitoes around the globe [6,7,8,9]. Given the limited arsenal of insecticides and increased resistance, there is a dire need to develop novel control strategies against *Ae. aegypti*. 

*Aedes aegypti* are primarily anautogenous, requiring a blood meal for every gonotrophic cycle. This dependence on blood meals makes them an ideal vector for multiple viral pathogens, including the dengue virus (DENV). The dengue transmission cycle begins when a female *Ae. aegypti* mosquito takes a blood meal from an infected host. The virus first infects and replicates in the mosquito midgut, eventually being released in the hemocoel. From this stage, the virus causes systemic infection in multiple mosquito tissues including the salivary gland. At this stage, the mosquito becomes infectious and can transmit DENV to a new human host when the mosquito takes another blood meal. The virus encounters multiple tissue barriers at each stage of the infection [10]. During the infection cycle in the vector, DENV interacts with multiple proteins that either positively or negatively affect the outcome of the infection [11,12]. One such reported protein is the *Ae. aegypti* cysteine-rich venom protein 379 (CRVP379, AAEL000379), which has been shown to interact with DENV and to be important for the establishment of infection [13]. 

The name cysteine-rich venom protein 379 was derived from similar cysteine-rich secreted proteins that were initially characterized from reptilian venoms. These proteins. broadly known as CRISPs, act as ion channel inhibitors and anticoagulants in reptile venoms [14]. CRISPs also play important roles in reproduction and immune systems in various organisms of the animal kingdom [15,16,17]. Despite being rich in cysteine residues, *Ae. aegypti* CRVP379 belongs to a broad group of proteins known as the cysteine-rich trypsin inhibitor-like proteins (TIL; PF01826.) since they contain a conserved cysteine-rich trypsin-like inhibitor domain (TIL). Apart from the conserved TIL domain, the proteins have diverged significantly. Even though these proteins possess a conserved TIL domain, very few have been experimentally shown to possess protease inhibitor activity. These proteins are widely distributed in the animal and plant kingdoms and play indispensable roles in multiple biological processes [18,19,20]. 

TIL domain-containing proteins remain understudied in vector mosquitoes. CRVP379 is a TIL domain-containing protein that has been studied in *Ae. aegypti* in the context of DENV infection. This protein was shown to be upregulated upon DENV infection, and RNA interference (RNAi)-mediated silencing of CRVP379 reduces DENV titers in *Ae. aegypti* [13]. 

In recent years, CRISPR-Cas9-based advances in genome editing have helped expand our understanding of the biological roles of many mosquito proteins. For example, CRISPR-Cas9-mediated knockout of both *Ae. aegypti* and *Anopheles gambiae* genes has more convincingly revealed their functions in mosquito biology than did RNAi-mediated gene silencing, which often resulted in a partial hylomorphic phenotype because of insufficient protein depletion [21,22,23]. 

In the present study we have analyzed the gene expression and effects of the CRVP379 protein in various tissues and life stages of *Ae. aegypti*. We also generated two lines of *Ae. aegypti* with different mutations in CRVP379, utilizing CRISPR-Cas9-mediated gene knockout to decipher the biological role of CRVP379 in *Ae. aegypti*. 

## 2. Results

As a first step towards understanding the role of CRVP379, we analyzed its DNA and protein sequences and compared them to other similar sequences available in the NCBI and VectorBase datasets. CRVP379 is located on chromosome 2 in the *Ae. aegypti* genome and has one intron and two exons. The gene encodes a 128-aa protein (13.7 kDa). BLAST results for CRVP379 showed multiple similar proteins present in *Ae. aegypti*, *Ae. albopictus*, and *Anopheles* and *Culex* genomes. CRVP379 showed sequence similarity to a wide variety of proteins, ranging from a small 82-aa protein from *Anopheles epiroticus* (AEPI007239) to a larger protein of 669 aa from *Culex quinquefasciatus* (CQUJHB008149.R12566). A phylogenetic analysis revealed that CRVP379 is closely related to the *Aedes albopictus* hypothetical protein (KXJ80742.1). Interestingly, in addition to mosquitoes, closely related proteins were also found in multiple spider species (Supplementary Figure S1A). 

CRVP379 contains a predicted signal peptide (1–19 aa) and a predicted TIL domain (23–79 aa), which consists of ten cysteine residues that form five disulfide bonds (1–7, 2–6, 3–5, 4–10 and 8–9, Supplementary Figure S1B). This arrangement is conserved in most of the proteins belonging to the TIL protein superfamily. All of the proteins with sequence similarity to CRVP379 (from the BLAST results) showed the presence of this conserved TIL domain. 

### 2.1. CRVP379 mRNA Abundance

To gain insight into the possible functions of CRVP379 in *Ae. aegypti* biology, we first analyzed its mRNA abundance at various life stages and in various tissues under a range of conditions. 

**Figure 1:**
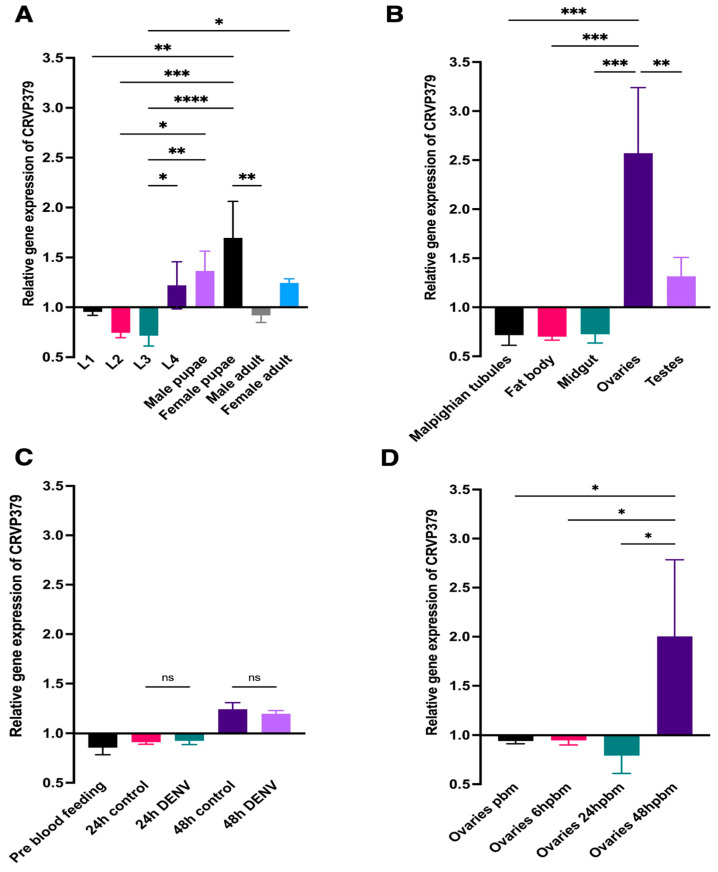
Relative gene expression of CRVP379 in different life stages and tissues of *Aedes aegypti*. (**A**) CRVP379 relative gene expression in the whole bodies of larval (L1–L4) and both male and female pupal and adult stages. (**B**) CRVP379 relative gene expression in various tissues of adult mosquitoes. (**C**) CRVP379 relative gene expression in the midgut before blood feeding and at 24 and 48 h after DENV infection. (**D**) CRVP379 relative gene expression in the ovaries before blood feeding and at 24 and 48 h after blood feeding. Means were compared with Tukey’s multiple comparisons test; * = *p* < 0.05, ** = *p* < 0.01, *** = *p* < 0.005, **** = *p* < 0.0001. Relative gene expression (RGE) was calculated by dividing the delta ct for an individual life stage or condition by the average delta ct for that experiment. ns—non-significant.

CRVP379 was expressed at low levels during the first three larval instars. Larval instar L3 had the lowest comparative CRVP379 mRNA abundance among the assayed life stages, having a relative gene expression (RGE) of 0.7166 ± 0.1046 (an RGE of 1.0 being the average RGE across all the life stages) (Figure 1A). CRVP379 mRNA abundance increased in the fourth instar, with a statistically significant increase in the RGE to between L3 and L4 (Figure 1A). When compared to the larval stages, the pupal stage in both males and females showed an elevated abundance of CRVP379 mRNA, with mean RGE values of 1.363 ± 0.1994 and 1.696 ± 0.3665, respectively (Figure 1A). There was a statistically significant increase in the CRVP379 RGE between the pupal stage and the first three larval instars in females. In adult mosquitoes, both adult males and females showed reduced gene CRVP379 expression as compared to their respective pupal stages. Male adults showed a lower CRVP379 RGE (0.9195 ± 0.07066) than did female adults (1.244 ± 0.04201, Figure 1A).

Next, we analyzed the CRVP379 mRNA abundance in various tissues of male and female adults. CRVP379 was expressed at very low levels in the female midgut, fat body, and Malpighian tubules, with RGEs of 0.7267 ± 0.09050, 0.7007 ± 0.03540, and 0.7166 ± 0.1046, respectively. Male testes showed the second highest RGE, 1.316 ± 0.1933. Female ovaries showed a significantly higher abundance of CRVP379 mRNA than did all the other assayed tissues (RGE 2.572 ± 0.6676, Figure 1B). 

CRVP379 was previously reported to play an important role in the midgut during DENV infection [13]. Therefore, we investigated the abundance of CRVP379 mRNA in the midgut at 24 and 48 h post-DENV infection. There was no significant difference in the CRVP379 mRNA abundance before blood feeding and 24 h after blood feeding in the midgut, regardless of infection status. CRVP379 mRNA abundance was significantly higher at 48 h after blood feeding than either before blood feeding or 24 h afterwards. However, this increase was seen in both the control and DENV-infected mosquitoes, with a significant difference between them (Figure 1C). These results indicate that CRVP379 transcription is regulated by blood feeding and not DENV infection. 

Since our expression analyses showed that CRVP379 was highly expressed in *Ae. aegypti* female ovaries, we sought to investigate the influence of blood feeding on the transcriptional regulation of ovarian CRVP379 by blood feeding. We saw no difference in the CRVP379 mRNA abundance between unfed and blood-fed (6 h post-blood feeding) females, with RGEs of 0.9399 ± 0.02709 and 0.9459 ± 0.04592, respectively. CRVP379 mRNA abundance decreased slightly at 24 h after blood feeding in the ovaries, with an RGE of 0.7922 ± 0.1814. However, this decrease was not statistically significantly different from the values for unfed females or at 6 h after blood feeding. Interestingly, at 48 h after blood feeding, the CRVP379 mRNA abundance levels increased significantly (RGE 2.003 ± 0.7814) in the ovaries when compared to the pre-feeding and the 6- and 24-h post-feeding time points (Figure 1D). 

### 2.2. CRVP379 Is Present in Ae. aegypti Ovaries but Not Secreted in the Hemolymph

**Figure 2:**
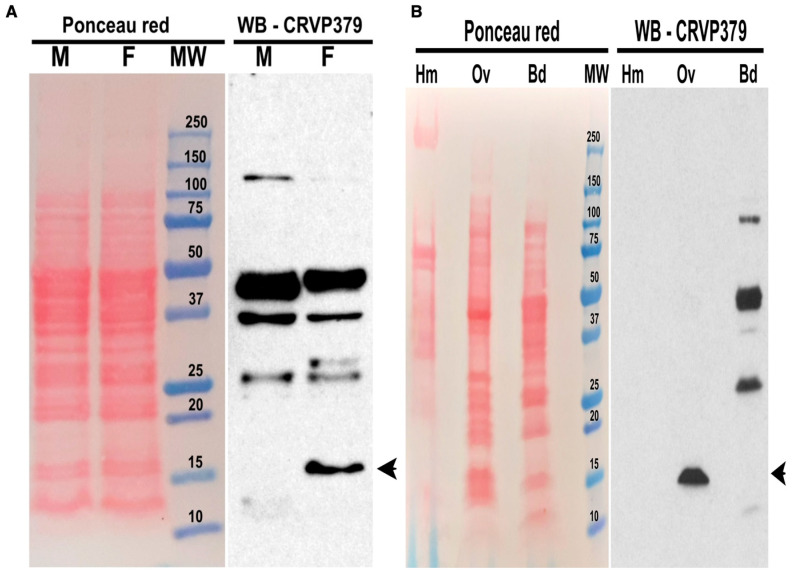
Western blot of CRVP379: the left panel shows protein staining with Ponceau red; the right panel shows western blotting with the CRVP379 antibody. In (**A**) M: male whole body; F: female whole body, MW: molecular weight marker. (**B**) Hm: female hemolymph; Ov: female ovaries; Bd: remaining female body without ovaries or hemolymph. Arrowhead—CRVP379.

We further analyzed CRVP379 protein abundance in *Ae. aegypti* mosquitoes with western blotting, utilizing a polyclonal antibody against CRVP379 protein. CRVP379 is a 128-aa, 13.7-kDa protein, and whole-body western blot analysis showed that CRVP379 is more abundant in adult female mosquitoes than in males (Figure 2A). The CRVP379 polyclonal antibody was not highly specific and also recognized other putative proteins, as indicated by the observance of multiple bands. Our mRNA abundance analysis showed that CRVP379 is highly expressed in the ovaries. Western blotting with proteins from female ovaries showed a high enrichment of CRVP379. The protein also has a predicted signal peptide, and we therefore investigated whether CRVP379 is secreted into the hemolymph. We did not detect CRVP379 in either the hemolymph or female bodies lacking ovaries, suggesting that CRVP379 is not secreted from the ovaries (Figure 2B). We also did not detect the presence of CRVP379 in female midguts (Supplementary Figure S2). Upon analyzing protein extracts from male testes, we observed non-specific binding to multiple proteins and one intense band at about 10 kDa, which was lower than the molecular size of CRVP379 (Supplementary Figure S2). This band may represent CRVP379 without its native signal peptide; however, the results of these western blots of the testes must be considered inconclusive because of the lack of specificity of the antibody available (Supplementary Figure S2). 

### 2.3. CRVP379 Gene Silencing Does Not Influence DENV Infection in the Midgut

**Figure 3:**
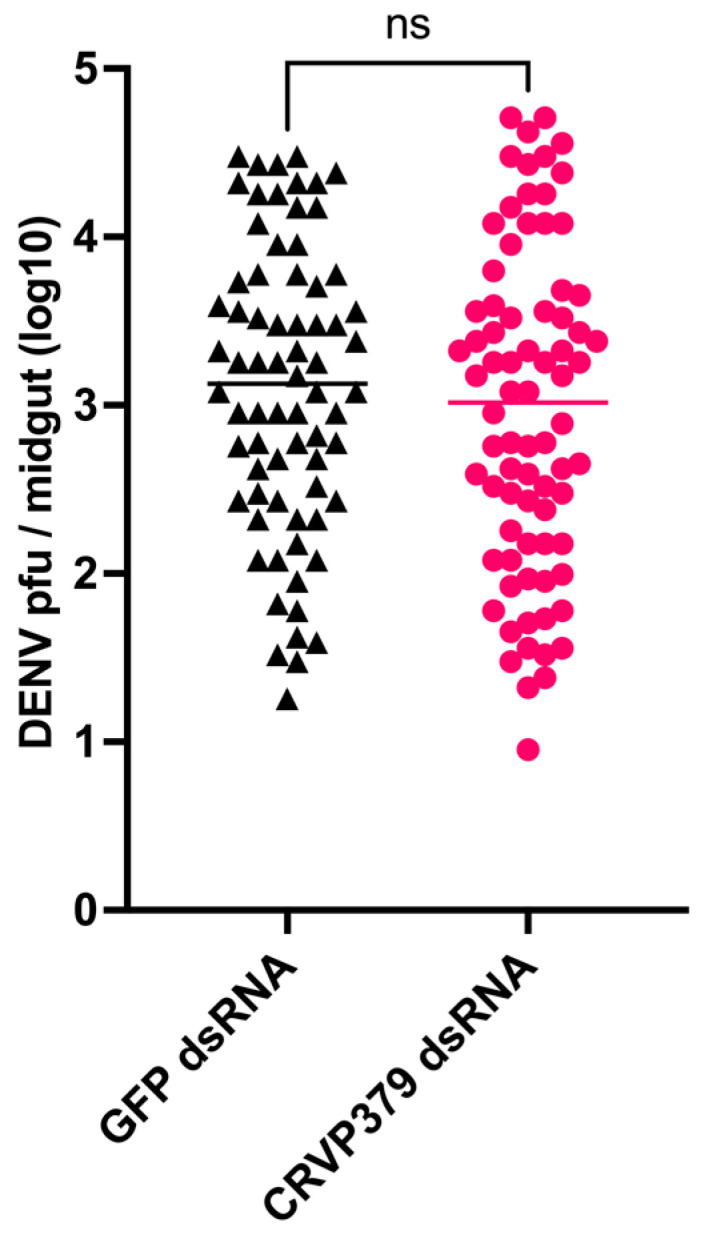
Effect of CRVP379 gene silencing on DENV titers in *Ae. aegypti* Liverpool midguts seven days after infection. Control mosquitoes injected with GFP dsRNA (*n* = 72), CRVP379 dsRNA injected misquotes (*n* = 78). ns—non-significant.

A previous study reported that silencing CRVP379 decreased DENV titers in the *Ae. aegypti* midgut [13]. Since we observed low CRVP379 mRNA and protein abundance in the midgut, we decided to retest the effect of CRVP379 silencing on DENV infection in the midgut. Three days after CRVP379 dsRNA injection, CRVP739 mRNA abundance was reduced by 50.71% to 70.53% when compared to the GFP dsRNA-injected controls. Our results showed that at seven days post-infection, the mean log DENV titer in the midgut of control mosquitoes injected with GFP dsRNA was 3.107 pfu/midgut (*n* = 72), and for CRVP379-silenced mosquitoes it was 2.966 pfu/midgut (*n* = 78). Even though this difference represented a slight reduction in DENV titers in the midgut upon silencing of the CRVP379 gene, this reduction was not statistically significant (Mann-Whitney test, *p* = 0.3484, Figure 3). There was also a slight reduction in DENV prevalence in CRVP379-silenced mosquitoes (59.40%) as compared to the GFP controls (65.49 %), although it also was not statistically significant (Fisher’s exact test, *p* = 0.3572). 

In the previous study the *Ae. aegypti* Rockefeller strain was used in gene silencing experiments, whereas here we used the Liverpool strain. Therefore, we also performed gene silencing experiments in the *Ae. aegypti* Rockefeller strain after DENV infection. As we observed for the Liverpool strain, we did not see any significant difference in the DENV titers in the midgut at four- or seven-days post-infection when the CRVP379 gene was silenced (Mann-Whitney test, *p* = 0.2645 for day four, *p* = 0.1239 for day seven; Supplementary Figure S3). 

### 2.4. Generation of CRVP379 Knockout Lines Using the CRISPR-Cas9 System

**Figure 4:**
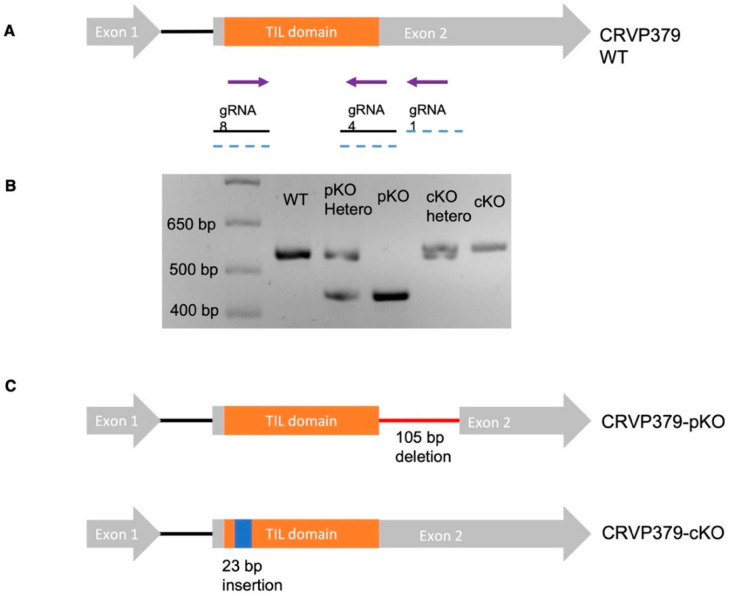
Schematic representation of CRISPR-Cas9-mediated knockout of CRVP379. (**A**) Guide RNAs used for embryo injection; guide RNAs highlighted with a dashed blue line were used together for injection 1; guide RNAs highlighted with a solid black line were used together for injection 2. (**B**) Agarose gel electrophoresis of PCR products of the CRVP379 gene with the CRVP Seq F/R primer set, from left to right: molecular weight ladder, wild type Liverpool, CRVP-pKO heterozygous, CRVP-pKO homozygous (105-bp deletion), CRVP-cKO heterozygous, CRVP-cKO homozygous (23-bp insertion). (**C**) Schematic representation of CRVP-pKO and CRVP-cKo mutations.

To gain further insight into the role of CRVP379 in the biology of *Ae. aegypti*, we generated knockout lines utilizing our previously established CRISPR-Cas9 gene-editing method [21]. We used the *Ae. aegypti* Exu-Cas9 line, which expresses Cas9 driven by an embryo-specific promoter [24]. We performed two independent rounds of embryo injections with two separate mixes of guide RNAs (gRNAs, Figure 4A). The first embryo injection experiment involved a gRNA mix of gRNA 8, 4, and 1, and the injection of a total of 427 eggs, yielding a hatching rate of 59.25%. The second experiment involved injection of a gRNA mix comprising gRNA 8 and gRNA 4. For the second experiment we injected 245 eggs and obtained a hatching rate of 33.87%. Mutations in the surviving G0 were assessed by PCR and Sanger sequencing. From embryo injection experiment 1 we recovered 24 males and 24 females from G0 that were analyzed for CRVP379 mutations by PCR. All of the mosquitoes tested showed two distinct bands on agarose gels, which proved to represent a deleted region of the gene (Figure 4B). From embryo injection experiment 2, we recovered 12 males and 12 females that were analyzed for CRVP379 mutations. A total of 14 mosquitoes (six males, eight females) showed two distinct PCR bands on agarose gels, which were shown to indicate insertions in the gene (Figure 4B). Putative mutants from G0 were outcrossed with virgin *Ae. aegypti* Liverpool mosquitoes, and after confirming the mutations in G1, we maintained and outcrossed two independent lines with *Ae. aegypti* Liverpool mosquitoes for five generations. After five generations, a loss of the Exu-cas9 cassette was confirmed by screening the larvae under red fluorescent light. Sequencing of CRVP379 fragments from these mosquitoes confirmed a 105-bp deletion between gRNA 1 and gRNA 4 in one line. This deletion was in-frame with the coding sequence toward the end of the TIL domain, so this line was therefore designated as a CRVP379-partial knockout (CRVP379-pKO). CRVP379-pKO was predicted to produce a mutant protein with a missing 35-aa region towards the C-terminus. This CRVP-pKO line had most of its predicted TIL domain intact, with only one cysteine and 5 aa missing from it (Figure 4B,C). 

Sequencing of CRVP379 from the second KO line confirmed a 23-bp insertion near gRNA 8. This insertion was a result of a duplication of a 23-bp sequence adjacent to gRNA8 (Figure 4B,C). This insertion was in the predicted TIL domain, and it disrupted the open reading frame of the gene; hence, this line was designated CRVP379 complete knockout (CRVP379-cKO). This line was predicted to produce a 90-aa mutant protein with minimal similarity to wild-type CRVP379 and no intact TIL domain. The CRVP-cKO line did not yield hatchable eggs in its homozygous state, so this line was maintained in a heterozygous state. The predicted mutant protein sequences from the CRVP-pKO and cKO lines are provided in Supplementary file S1. 

### 2.5. CRVP379 Is Important for Follicular Morphology in Ae. aegypti Ovaries

**Figure 5:**
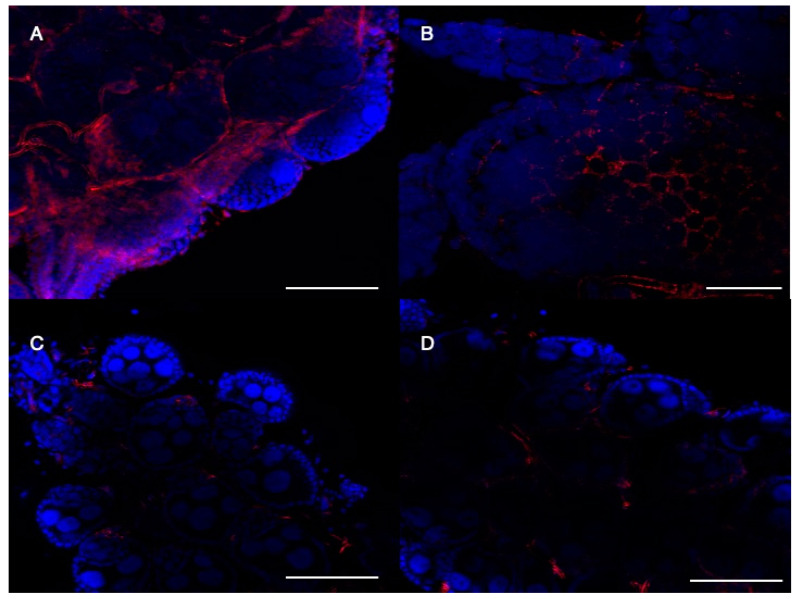
Immunofluorescent detection of CRVP379 in the ovaries of *Ae. aegypti*. Anti-CRVP379 antibody in red, DAPI (nuclei) in blue. (**A**) *Ae. aegypti* Liverpool ovaries, 20× magnification. (**B**) *Ae. aegypti* Liverpool ovaries, 63× magnification, in both (**A**,**B**). CRVP379 can be seen in the follicular epithelial cells. (**C**) CRVP-pKO ovaries, 20× magnification. (**D**) CRVP-cKO ovaries, 20× magnification. In both CRVP-pKO and CRVP-cKO ovaries, a clear localization of CRVP379 was not observed. Images are representative images of ovaries from each line. Images were obtained by confocal microscopy; Z-slides were stacked to get a single projection. Bar scale (**A**,**C**,**D**) = 100 μm, (**B**) = 20 μm.

Next, we performed Western blots with protein extracts from adult females of both the mutant pKO and cKo lines to detect CRVP379. We observed a significant reduction in detectable CRVP379 protein in both the pKO and cKO lines (Supplementary Figure S4). To investigate whether CRVP379 was present in the ovaries, we performed immunofluorescent staining on dissected ovaries from wild-type and mutant lines. In the wild-type mosquitoes, CRVP379 was clearly present in the follicular cells (Figure 5A), and specifically in the periphery of these cells (Figure 5B). CRVP379 was not detected in either the pKO or cKO lines in the ovarian follicular cells (Figure 5C,D). Both western blot analysis and confocal microscopy confirmed the loss of CRVP379 from the mutant lines. We also investigated the presence of CRVP379 in male testes by immunofluorescent staining. However, CRVP379 could not be clearly localized in the testes because of the presence of non-specific background fluorescence in both the wild-type and mutant lines (Supplementary Figure S5). The confocal microscopy for testes is in accordance with our western blot results.

Our microscopy studies also revealed striking differences in the general morphology of the follicles between wild-type and mutant mosquitoes. In the wild-type mosquitoes, the follicles had a well-defined follicular epithelial cell lining, whereas in both mutant lines the follicular cell lining was less defined and showed an abnormal morphology (Supplementary Figure S6). The size of the follicles also appeared to be smaller in the mutant lines than in the wild-type mosquitoes. Using the ImageJ Fiji software (area in pixels), we confirmed that the CRVP379-cKO line had significantly smaller follicles than did either the wild-type or the CRVP379-pKO line (Supplementary Figure S6).

### 2.6. CRVP379 Plays an Important Role in the Male and Female Reproductive Systems

**Figure 6:**
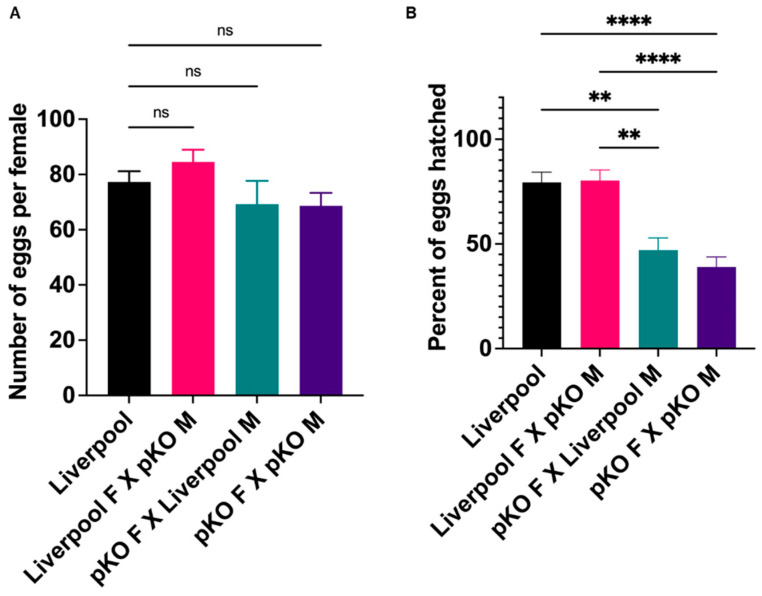
(**A**) Number of eggs laid per female with various crosses of the CRVP-pKO line; M = male, F = female. (**B**) Percentage of eggs hatched from various crosses of CRVP-pKO. Statistically significant *p* values are depicted by an asterisk (*); ** = *p* < 0.006, **** = *p* < 0.0001, error bars represent standard error of mean (Dunn’s multiple comparisons test, ns—non-significant).

As described above, CRVP379 was highly expressed in the ovaries, and the CRVP379-pKO and cKO lines showed abnormal follicular morphology. To investigate the role of CRVP379 in *Ae. aegypti* reproductive biology, we compared the fecundity of the CRVP379 mutant lines to wild-type mosquitoes. For this purpose, we performed crosses with both female and male wild-type Liverpool and CRVP-pKO males and females, respectively. For the pKO line, there was no significant difference in the number of eggs laid by female mosquitoes irrespective of the genotype of the male (Figure 6A). When we compared the hatching rate, we saw no difference between wild-type Liverpool male-female crosses (mean hatching rate, 79.38%) and wild-type female-CRVP379-pKO male crosses (mean hatching rate, 80.30%). These data indicate that CRVP-pKO males are reproductively comparable to Liverpool wild-type males (Figure 6B). However, in the case of females, CRVP379-pKO females showed a reduced hatching rate irrespective of the male genotype with which they were crossed. CRVP379 pKO females crossed with wild-type Liverpool males had a mean egg hatching rate of 47.01%, which was significantly lower than that for the Liverpool line or for crosses between Liverpool females and CRVP-pKO males (Dunn’s multiple comparison test, *p* < 0.006). Crossing of CRVP379-pKO females with CRVP-pKO males produced an even lower mean hatching rate of 38.97% (Dunn’s multiple comparison test) when compared to Liverpool crosses and Liverpool female-CRVP-pKO male crosses (*p* < 0.0001, Figure 6B). A simplified graphical version of various CRVP-pKO crosses is presented in the supplementary files (Supplementary Figure S7A). This result suggests that even a partial deletion of CRVP379 affects female reproductive capabilities, and the WT male genotype is unable to rescue this phenotype.

**Figure 7:**
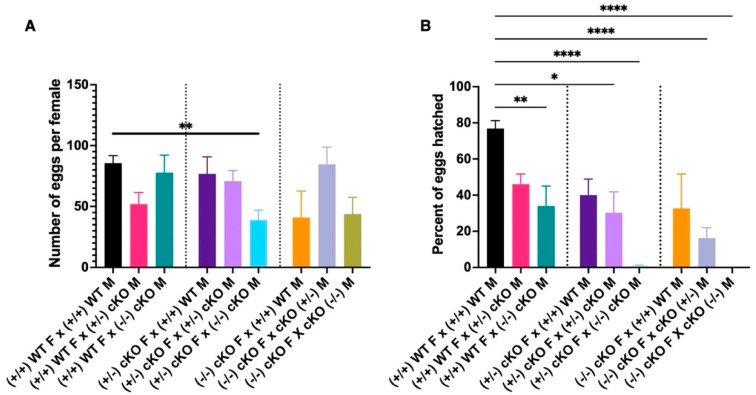
(**A**) Number of eggs laid per female for various crosses of the CRVP-cKO line. M = male, F = female. (**B**) Percentage of eggs hatched from various crosses of CRVP-cKO. Statistically significant *p* values are depicted with an asterisk (*); * = *p* < 0.05, ** = *p* < 0.007, **** = *p* < 0.0001, (Dunn’s multiple comparisons test), error bars represent standard error of mean. WT Liverpool +/+, CRVP379-cKO heterozygous genotype +/−, CRVP379-cKO homozygous genotype −/−.

For the CRVP-cKO line we were unable to maintain a homozygous genotype. Multiple crosses with CRVP-cKO homozygous mosquitoes yielded eggs that did not hatch (confirmed by leg PCR and sequencing). For this reason, every single mosquito had to be genotyped by leg PCR and sequencing before any experiment. Given its hampered reproduction, the CRVP-cKO line was maintained as a heterozygous genotype. Crosses between heterozygotes yielded wild-type, homozygous, and heterozygous mosquitoes. To assess the effect of complete CRVP379 knockout on male and female fecundity, we set up multiple crosses with wild-type, heterozygous, and homozygous mosquitoes. All but one of the CRVP-cKO crosses showed a significant difference from the wild-type in the number of eggs laid by an individual female. Only crosses between CRVP370-cKO heterozygous females and CRVP-cKO homozygous males produced significantly lower numbers of eggs when compared to the Liverpool strain (Dunn’s multiple comparisons test, *p* = 0.0066, Figure 7A). However, we did see significant differences in hatching rates with the various CRVP-cKO crosses. When wild-type Liverpool females were crossed with CRVP-cKO homozygous males, there was a significant reduction in the hatching rate (33.94%, *p* = 0.0072) when compared to the Liverpool WT mosquitoes (76.84% hatching rate, Figure 7B). When CRVP-cKO heterozygous females were crossed with wild-type Liverpool males, there was no difference in the hatching rate. However, when CRVP-cKO heterozygous females were crossed with either heterozygous or homozygous males, we again observed a significant reduction in hatching rate when compared to the wild-type Liverpool crosses (Dunn’s multiple comparisons test, *p* = 0.0317 and *p* < 0.0001 respectively; Figure 7B). When CRVP-cKO homozygous females were crossed with wild-type Liverpool males, the hatching rate was comparable to that for the wild-type Liverpool crosses. Like the previous results, when homozygous females were crossed with heterozygous males, a significant reduction in the hatching rate was observed (Dunn’s multiple comparisons test, *p* < 0.0001; Figure 7B). For homozygous CRVP-cKO crosses, we were unable to obtain viable eggs from the crosses. None of eggs laid by the CRVP-cKO homozygous cross females hatched (Figure 7B). A simplified graphical version of various CRVP-cKO crosses is presented in the supplementary files (Supplementary Figure S7B). Preliminary observations indicate that the eggs from this cross had a collapsed chorion, typical of nonviable eggs previously reported from *Ae. aegypti* [25]. These experiments clearly show that CRVP379 plays an important role in both the male and female reproductive systems, since crosses involving both males and females from the CRVP-cKO line had a significantly lowered egg hatching rate.

### 2.7. CRVP379 Does Not Influence DENV Infection in the Midgut

**Figure 8:**
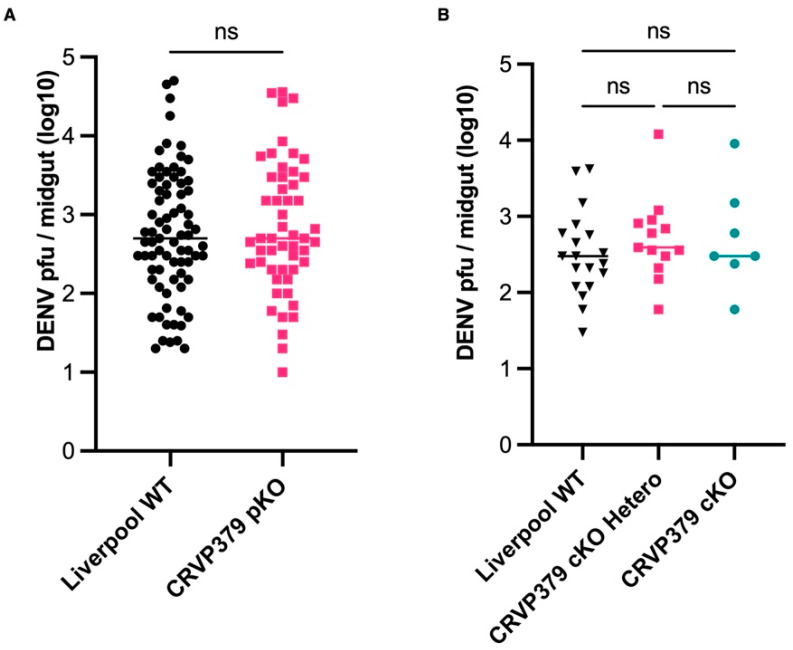
DENV titers in *Ae. aegypti* midguts at 7 days after infection, as determined by plaque assay. (**A**) CRVP-pKO compared with wild-type Liverpool mosquitoes. (**B**) CRVP-cKO heterozygous and homozygous mosquitoes compared with wild-type Liverpool mosquitoes. (Dunn’s multiple comparisons test, ns = not statistically significant).

Next, we investigated whether the CRVP-pKO and cKO lines showed altered permissiveness to DENV infection in the midgut at seven days after infection, but the CRVP-pKO and wild-type Liverpool mosquitoes did not differ in their DENV infection prevalence (Liverpool 80.21%, CRVP-pKO 82.54%, Fisher’s exact test, *p* = 0.8366). There was also no significant difference in DENV infection intensity (titer) in the midgut at seven days after infection (Mann-Whitney test, *p* = 0.6501; Figure 8A). Liverpool mosquitoes had a mean log DENV titer of 2.754, while CRVP-pKO mosquitoes had a mean log DENV titer of 2.832. For the CRVP-cKO line, there was no significant difference in prevalence among the wild-type Liverpool, CRVP-cKO heterozygous, and CRVP-cKO homozygous lines (Fisher’s exact test, *p* > 0.999). There was also no significant difference in DENV infection intensity among these lines. The mean log DENV titer in the midgut at seven days after infection for the Liverpool, CRVP-cKO heterozygous, and CRVP-cKO homozygous lines was 2.505, 2.700, and 2.717, respectively (Figure 8B). It should be noted that the sample size for the infection experiments with CRVP-cKO were relatively smaller because of the difficulty in maintaining this line. Thus, these data corroborate our previous gene silencing experiments showing that CRVP379 does not serve as a DENV host or restriction factor.

### 2.8. CRVP-pKO Females Have a Significantly Reduced Lifespan

Next, we asked whether the CRVP379 mutations influenced the lifespan of male and female mosquitoes. Because of the limited number of mosquitoes available from the CRVP-cKO line, we were unable to perform statistically meaningful lifespan experiments with this line. All of the lifespan experiments were carried out with the CRVP-pKO line. The male CRVP-pKO mosquitoes were not significantly different in lifespan than the wild-type mosquitoes. The median lifespan for the Liverpool males was 28 days, and that of the CRVP-pKO males was 29 days (log-rank Mantel-Cox test, *p* = 0.4545, Supplementary Figure S8A). The CRVP-pKO females displayed a significantly reduced lifespan when compared to the Liverpool wild-type mosquitoes. The median life span of the Liverpool females was 44 days, whereas the CRVP-pKO mosquitoes had a median life span of only 21 days (log-rank Mantel-Cox test, *p* < 0.0001, Supplementary Figure S8B). This result demonstrates that even a partial deletion in CRVP379 outside of its TIL domain has a significant impact on female lifespan but no effect on male lifespan.

## 3. Discussion

Here we sought to understand the role of cysteine-rich venom protein 379 (CRVP379) in *Ae. aegypti* biology by utilizing a CRISPR-Cas9-mediated gene knockout approach. Our study showed that CRVP379 is highly expressed in male testes and female ovaries. We also observed that CRVP379 expression is upregulated in the ovaries 48 h after blood feeding. Immunohistological assays localized CRVP379 to the periphery of follicular epithelial cells in *Ae. aegypti* ovaries. We further showed that RNAi-mediated gene silencing and gene knockout of CRVP379 did not have an impact on DENV infection in *Ae. aegypti* mosquitoes. Experiments utilizing our CRISPR-Cas9-generated mutant lines suggested that CRVP379 can be at least partially functional when the deletion occurs outside the TIL domain. A CRVP379-pKO line with a partial in-frame deletion that left most of the TIL domain intact showed no difference with regards to male lifespan or male reproductive capabilities. However, female mosquitoes from this line had a significantly lower lifespan and egg hatching rate. When CRVP379 was completely inactivated in our CRVP379-cKO line, both male and female reproduction were severely hampered. We observed a smaller follicular size in both CRVP379 mutant lines. Overall, our study highlights the importance of CRVP379, a TIL domain-containing protein, in *Ae. aegypti* reproduction and possibly in other processes that regulate the female life span.

Even though TIL domain-containing proteins are distributed throughout the animal kingdom, very few proteins from insects have been reported or studied. The endoparasitoid wasp *Cotesia vestalis* has been shown to secrete a TIL protein from its teratocytes that blocks its host’s melanization activity [26]. A cysteine rich TIL domain-containing protein from *Bombus ignitus* bumblebee venom has been shown to possess protease inhibitor and antifibrinolytic activity [27]. Similar cysteine-rich TIL domain-containing proteins from *Bombyx mori* have been shown to possess antifungal activity and effectively inhibit multiple microbial proteases [28,29]. These studies indicate that proteins with a conserved TIL domain are likely to have diverse tissue- and life stage-specific roles in different insects.

Phylogenetic analysis of *Ae. aegypti* CRVP379 has revealed multiple TIL domain-containing proteins in both *Anopheline* and *Culicine* mosquitoes. These proteins showed a high degree of conservation in the TIL domain but were otherwise diverse in terms of sequence and length. In humans, a TIL domain-containing protein, Von Willebrand factor, is translated as a massive 2818-aa protein and plays a key role in blood clotting [30,31]. At the same time, in the frog *Lepidobatrachus laevis*, a small 55-aa peptide containing the same TIL domain has protease-inhibiting activity [32]. These examples highlight the fact that despite the presence of a conserved TIL domain, there is functional and spatial diversity in TIL-containing proteins.

Our gene expression analysis indicated an increased expression of CRVP379 during the pupal stage. These results are similar to those from a previous study in the cotton bollworm *Helicoverpa armigera*, in which a TIL domain-containing protein, HaTIL2, was highly expressed in the pupal stage [33]. Multiple *Ae. aegypti* and *Ae. albopictus* trypsins are known to play an important role during the pupal stage [34,35,36]. It is likely that CRVP379-like trypsin inhibitors may similarly be playing an important role in trypsin regulation during the pupal stage. We also observed CRVP379 expression in male testes and high expression in female ovaries. These data are in accord with the *Ae. aegypti* atlas and *Ae. aegypti* developmental transcriptome by Akbari et al., in which CRVP379 is shown to be enriched in the ovaries and testes [37,38]. This finding was further corroborated by our western blotting and immunostaining experiments. We found CRVP379 to be localized to the periphery of the follicular epithelial cells. However, despite the presence of a signal peptide, we did not observe CRVP379 to be secreted into the hemolymph. This observation raises the possibility that the signal peptide plays a role in localizing the protein around the follicular epithelial cells rather than in extracellular secretion. The expression of CRVP379 was significantly increased in the ovaries at 48 h after blood feeding. All of these findings suggest that CRVP379 plays a key role in *Ae. aegypti* reproduction. An increase in the expression during pupal stages and in the ovaries after blood feeding strongly suggests a hormonal regulation of CRVP379. During these two stages, various hormones, such as 20E and juvenile hormone (JH) regulate multiple genes in mosquitoes [39]. Juvenile hormone also plays a key role in previtellogenic ovarian development [40,41]. A chymotrypsin-like serine protease in *Ae. aegypti* has been shown to be regulated by JH [42]. It would be interesting to study the possibility of hormonal regulation of CRVP379 in more detail in the future.

Interestingly, in contrast to the previously reported findings by Londono-Renteria et al., we did not detect a significant CRVP379 presence in the midgut [13]. We also did not observe any changes in CRVP379 gene expression after DENV infection. Similarly, gene silencing experiments also had no effect on DENV titers in two different *Ae. aegypti* lab strains. Because we did not observe CRVP379 in the midgut, it is unlikely to play a role in the DENV infection of that tissue. However, our results are not perfectly comparable to the study by Londono-Renteria et al. because of inherent differences in methodology and a lack of sufficient information concerning experimental procedures, such as their primer sequences used for gene expression and silencing [13].

Other than the signal peptide, the only notable feature of CRVP379 is the TIL domain, although its importance had never been experimentally validated. The importance of the C-terminus of this protein, which does not have any notable features, is also unknown. We were able to create two distinct CRVP379 mutant lines, one with a mutation inside and one outside the TIL domain. The CRVP379 partial knockout line (CRVP379-pKO) had a mutation outside the TIL domain and could still produce a 93-aa protein with most of its TIL domain intact. The partial knockout line (CRVP-pKO) did not show any altered permissiveness to DENV infection in the female midgut or effects on male lifespan or male reproduction. However, females from this line had a significantly shorter lifespan and reduced fecundity. These results raise two possibilities: (a) the CRVP379 residues between aa76 and aa110 play an important role in female *Ae. aegypti* but not in males; and (b) CRVP-pKO produces a completely inactive product, and CRVP379 does not play a role in either male reproduction or male lifespan. If the latter hypothesis were true, the other mutant line with a complete inactivation CRVP379-cKO should have shown a similar phenotype. However, the CRVP-cKO line had severe defects in both male and female reproduction. In fact, we were unable to maintain a homozygous knockout line because none of the eggs from this cross hatched. We also observed that CRVP-cKO homozygous males and females could produce viable eggs if the other partner had a wild-type CRVP379 genotype. This result suggests that CRVP379 may be important for fertilization and/or embryonic development. Localization of CRVP379 to the male reproductive system was not possible with our polyclonal antibody, since it bound to multiple proteins (i.e., was nonspecific). A more specific monoclonal antibody is likely to shed light on the localization of CRVP379 in the male reproductive tissues. In the case of both mutant lines, we observed smaller follicles in the females when compared to wild-type mosquitoes, with the cKO line showing the smallest follicles. Localization of CRVP379 to the follicular epithelial cells further supports a role for CRVP379 in the *Ae. aegypti* female reproductive system. These results are similar to another study carried out in the brown plant hopper *Nilaparvata lugens*, in which an inter-alpha-trypsin inhibitor has been shown to play an important role in reproduction and ovarian development [43].

It should be noted that it was challenging to maintain the CRVP mutant lines. The CRVP-pKO line demonstrated a very short lifespan and reduced fecundity, and the CRVP-cKO line could not be maintained as a homozygous genotype. To study the CRVP379-cKO line, heterozygous mosquitoes were crossed to obtain different genotypes of CRVP-cKO. Each mosquito among the progeny was then genotyped by leg PCR and the sequencing of the PCR product. Some of the progeny had to be utilized for maintenance of the line, and this necessity effectively limited the experiments we were able to perform with this line. Alternative strategies such as the use of conditional knockouts might permit a more comprehensive study of CRVP379.

Finally, we showed that CRVP379 does not play a role in DENV infection in the midgut, given that our data showed that both the pKO and cKO lines had the same DENV titer in the midgut at seven days after infection. Our gene expression analysis, gene silencing experiments, and DENV infection assays in our mutant lines comprehensively show that CRVP379 does not play a role in DENV infection.

Our study highlights the role of TIL domain-containing proteins in *Ae. aegypti* reproductive biology. We also show that certain residues in CRVP379 may play different roles in male and female mosquitoes. Given its importance in the biology of *Ae. aegypti* mosquitoes, which are key vectors of many viral diseases, CRVP379 deserves to be evaluated more fully in terms of its potential usefulness in developing novel mosquito population control strategies.

## 4. Materials and Methods

### 4.1. Ethics Statement

This study was carried out in strict accordance with the recommendations in the Guide for the Care and Use of Laboratory Animals of the National Institutes of Health and the Animal Care and Use Committee of the Johns Hopkins University (Permit Number: M006H300). Mice were only used for mosquito rearing as a blood source, according to approved protocol. Commercial anonymous human blood was used for DENV infection assays in mosquitoes, and informed consent was therefore not applicable. The Institutional Animal Care and Use Committee (IACUC) approved the protocol.

### 4.2. Mosquito Rearing

*Ae. aegypti* Liverpool, Rockefeller, and Exu-Cas9 strains were used in this study. *Ae. aegypti* Exu-Cas9 strain was a gift from Omar Akbari (University of California San Diego). All of the mosquitoes were maintained on a 10% sucrose solution at 27 °C and 80% relative humidity with a 14:10 h light-dark cycle in the Johns Hopkins Malaria Research Institute’s insectary.

### 4.3. Cell Culture

*Ae. albopictus* cells C6/36 (ATCC CRL-1660) were cultured in MEM (Gibco) supplemented with 10% FBS, 1% L-glutamine, 1% non-essential amino acids, and 1% penicillin/streptomycin. Cells were maintained at 32 °C with 5% CO_2_. Baby hamster kidney cells (BHK-21, ATCC CCL-10) were cultured in DMEM (Gibco) supplemented with 10% FBS, 1% L-glutamine, 1% penicillin/streptomycin, and 5 µg/mL Plasmocin (Invivogen, San Diego, CA, USA). Cells were maintained at 37 °C with 5% CO_2_.

### 4.4. Dengue Virus

DENV2 strain New Guinea C (NGC) was use in the study. The virus was propagated in C6/36 cells and stored at −80 °C. The virus titer was determined by plaque assay on BHK21 cells.

### 4.5. dsRNA Synthesis

Total RNA was extracted from a pool of five one-week-old *Ae. aegypti* Liverpool and Rockefeller females using the Trizol RNA extraction method. cDNA was synthesized from the total RNA using an oligo dT primer with the MMLV reverse transcriptase kit. Total cDNA was used to amplify *Ae. aegypti* CRVP379 (AAEL000379) using the primer set CRVP-RNAi-F and CRVP-RNAi-R (Supplementary Table S1). GFP gene was amplified using primer set GFP-RNAi-F and GFP-RNAi-R with GFP plasmid as a template (Supplementary Table S1). Amplified PCR products were purified using a Zymo DNA concentrator and cleanup kit. A total of 1 µg of PCR product was used to synthesize dsRNA using a NEB T7 HiScribe kit according to the manufacturer’s instructions. dsRNA was purified using isopropanol-sodium acetate precipitation. dsRNA was dissolved in sterile distilled water, and the concentration was measured with a Nanodrop spectrophotometer. The final concentration of the dsRNA was adjusted to 3 µg/µL.

### 4.6. Gene Silencing

One-week old female *Ae. aegypti* mosquitoes (Liverpool and Rockefeller) were anesthetized on a cold block and intrathoracically injected with 69 nL of 3 µg/µL dsRNA (either CRVP379 or GFP as a control). *Ae. aegypti* Liverpool and Rockefeller females were injected with dsRNA generated from the same strain. A pool of five females was collected at 72 h post-injection prior to infection with DENV2. RNA was extracted from this pool to quantify gene silencing efficiency.

### 4.7. Gene Expression

*Ae. aegypti* (Liverpool) mosquitoes were used to analyze the gene expression of CRVP379 at various life stages and in various tissues. Ten L1-L4 larvae, five male and five female pupae, and five male and five female adults were collected in Trizol and used for RNA extraction. The midgut, fat bodies, ovaries, and Malpighian tubules were dissected out and collected from a total of 10 one-week-old females. Testes were collected from a total of 20 one-week-old males. To study the CRVP379 gene expression in the ovaries after blood feeding, ovaries from 10 females were collected before blood feeding and at 6, 24, and 48 h after blood feeding. To quantify CRVP379 gene expression in the midguts after DENV2 infection, 20 female midguts were dissected and collected prior to DENV infection, and at 24 and 48 h after DENV infection. All of the tissues were collected in Trizol, and RNA was isolated using the Trizol extraction method. All of the experiments were carried out independently in triplicate. Gene expression was measured using TaqMan™ Universal Master Mix with CRVP qPCR primers. The *Ae. aegypti* S17 gene was used as a housekeeping gene control. Relative gene expression (RGE) was calculated by dividing the delta ct for an individual life stage or condition by the average delta ct for that experiment.

### 4.8. Generation of CRVP379 Knockout Lines

To generate CRVP379 knockout lines, the CRISPR-Cas9-mediated knockout method using the *Ae. aegypti* Exu-Cas9 line was utilized. *Ae. aegypti* Exu-Cas9 expresses Cas9 driven by an embryo-specific promoter. Guide RNAs targeting *Ae. aegypti* CRVP379 were designed using the CHOPCHOP web tool [44]. Guide RNAs were synthesized in vitro according to the previously described protocol using a gene-specific forward primer with a T7 promoter and a common reverse primer (Supplementary Table S1).

### 4.9. Embryo Injections

Early embryos (within 30 min of egg laying) of *Ae. aegypti*-Exu-Cas9 were injected with 100 ng/µL of each guide RNA. Embryos were injected independently with two or three different guide RNA mixes (Supplementary Table S1), as described previously. The surviving G0 adults were sexed, and mutations were confirmed by leg PCR followed by sequencing with CRVP primers (Supplementary Table S1).

### 4.10. Leg PCR

A quarter of a hind leg of a four-day-old adult mosquito was dissected and suspended in 19 µL of squash buffer and 1 µL of proteinase K (Promega). The legs were incubated at 56 °C for 30 min, followed by incubation for 10 min at 95 °C. Five microliters of the above solution was then used for PCR with NEB OneTaq 2X master mix in a 25-µL reaction with the primer set. PCR products were sequenced, and gene sequences and peaks were compared to the *Ae. aegypti* Liverpool CRVP379 gene sequence. Mosquitoes with mutations were individually outcrossed with the *Ae. aegypti* Liverpool strain. After five generations of outcrossing with the Liverpool strain, mutant lines were in-crossed, and homozygous mutant lines were obtained. Two *Ae. aegypti* lines with a different mutation in the CRVP379 gene were generated. One line was designated as CRVP partial knockout (CRVP-pKO, 105-bp deletion), and the other was designated as CRVP379 complete knockout (CRVP-cKO, 23-bp insertion).

### 4.11. Line Maintenance, Fecundity, and Life Span

The CRVP379 complete knockout line (CRVP379-cKO) was maintained as a heterozygous genotype. For each generation, mosquitoes were genotyped by leg PCR as described above. Multiple crosses were set up to include both males and females of wildtype CRVP379, heterozygous CRVP379-cKO mutant, and homozygous CRVP379-cKO genotypes. Female mosquitoes were fed on anesthetized mice, and fully engorged females were selected for fecundity experiments. Three days after blood feeding, individual females were added to 50-mL conical tubes lined with moist filter paper. Individual females were allowed to lay eggs in one tube for 48 h. After 48 h, the filter papers were removed, and the eggs were allowed to dry for five days. Five days later, the eggs were hatched in sterile hatching broth, and the larvae were counted three days later. The life spans of the CRVP379-pKO and Liverpool parental lines were measured as described previously. In brief, male and female mosquitoes were sexed at the pupal stage. A total of 30 virgin males and females were maintained in a paper cup and given 10% sucrose solution on a piece of cotton. Mosquito mortality was measured every day.

### 4.12. Immunoblotting

Mosquitoes (either complete mosquitoes or individual tissues) were lysed in 40 μL of RIPA Buffer (Sigma, St. Louis, MO, USA, R0278) containing protease inhibitor cocktail (Complete, EDTA-free; Roche, Basel, Switzerland). Protein concentrations were measured using a Micro BCA Protein Assay Kit (Thermo Scientific, Waltham, MA, USA, # 23235), and the concentration of the samples was adjusted to 5 μg/μL. Bolt LDS Sample buffer (Invitrogen, Waltham, MA, USA, NP0007) and Bolt/NuPAGE reducing agent load (Invitrogen, NP0009) were added, and samples were heated at 100 °C for 5 min. Samples (30 μg of protein) were run in Tris-glycine gel, 4–20% (Invitrogen, XP04205BOX) at a constant 25 mA for 75 min. Proteins were electro-transferred to a nitrocellulose membrane (0.2-μm pore size; BIO-RAD, 1704271) using a Trans-Blot Turbo Transfer System (BIO-RAD, Hercules, CA, USA, 1704150) at 2.5 mA for 7 min. The membrane was stained with Ponceau S staining solution (Cell Signaling Technology, Danvers, MA, USA, 54803S) to corroborate the quality of transfer, followed by three washes with PBS for 10 min each. The membrane was blocked with Blocker Solution (blotting-grade blocker; BIO-RAD, 170604) at 5% on PBS for 60 min at room temperature (RT). The membrane was incubated overnight with 0.5× of blocker solution containing 1:2000 of rabbit anti-*Aedes aegypti* CRVP379 antibody (Boster Biological Technology, Pleasanton CA, USA, Catalog # DZ41086) at 4 °C. CRVP379 antibody at a dilution of 1:2000 was observed to be optimum for western blots. The membrane was washed three times with PBST (PBS with 0.2% Tween 20), then incubated with 0.5× of blocker solution containing 1:10,000 Anti-Rabbit IgG, HRP-linked Antibody (Cell Signaling Technology, 7074) for 60 min at RT, washed again, and developed using ECL Prime Western Blotting Detection Reagent (GE Healthcare, Chicago, IL, USA, RPN2232) and High Performance Chemiluminescence Film (GE Healthcare).

### 4.13. Immunohistochemical Staining and Microscopy

*Ae. aegypti* Liverpool, CRVP379-pKO, and CRVP379-cKO males and females were cold-anesthetized on ice. Ovaries and testes from four-day old mosquitoes were dissected using sharp forceps and entomological needles. The ovaries were stained following a method previously described [45]. CRVP379 antibody was used at a dilution of 1:200, and AlexaFluor568-goat anti-rabbit antibody (Life Technologies, A11011) was used at a dilution of 1:200. Samples were mounted using Fluoromount-G with DAPI (Invitrogen, 00-4959-52) and examined with a Zeiss LSM 1710 confocal microscope. Images were analyzed with ImageJ Fiji software [46]. The Z-stack (all slides) data sets were analyzed and presented as a single 2D projection. Brightfield and DAPI stained ovaries were also observed under a LEICA DM2500 microscope, and images were captured with a DFC310 FX camera. Three ovaries from each line were used to measure the follicular cell size using ImageJ Fiji software, License GPLv3+, Release 2.5.0.

## Data Availability

Not applicable.

## Supplementary Materials

The following supporting information can be downloaded at: https://www.mdpi.com/article/10.3390/ijms23147736/s1.
