# Supplementary material for: Trypsin-like Inhibitor Domain (TIL)-Harboring Protein Is Essential for Aedes aegypti Reproduction
Source: Int J Mol Sci. 2022 Jul 13;23(14):7736. doi: 10.3390/ijms23147736 (PMC9319116; doi:10.3390/ijms23147736)
Supplement: Supplementary file 1 [file ijms-23-07736-s001.zip › ijms-1784779-supplementary v4.pdf]

SUPPLEMENTARY FIGURES

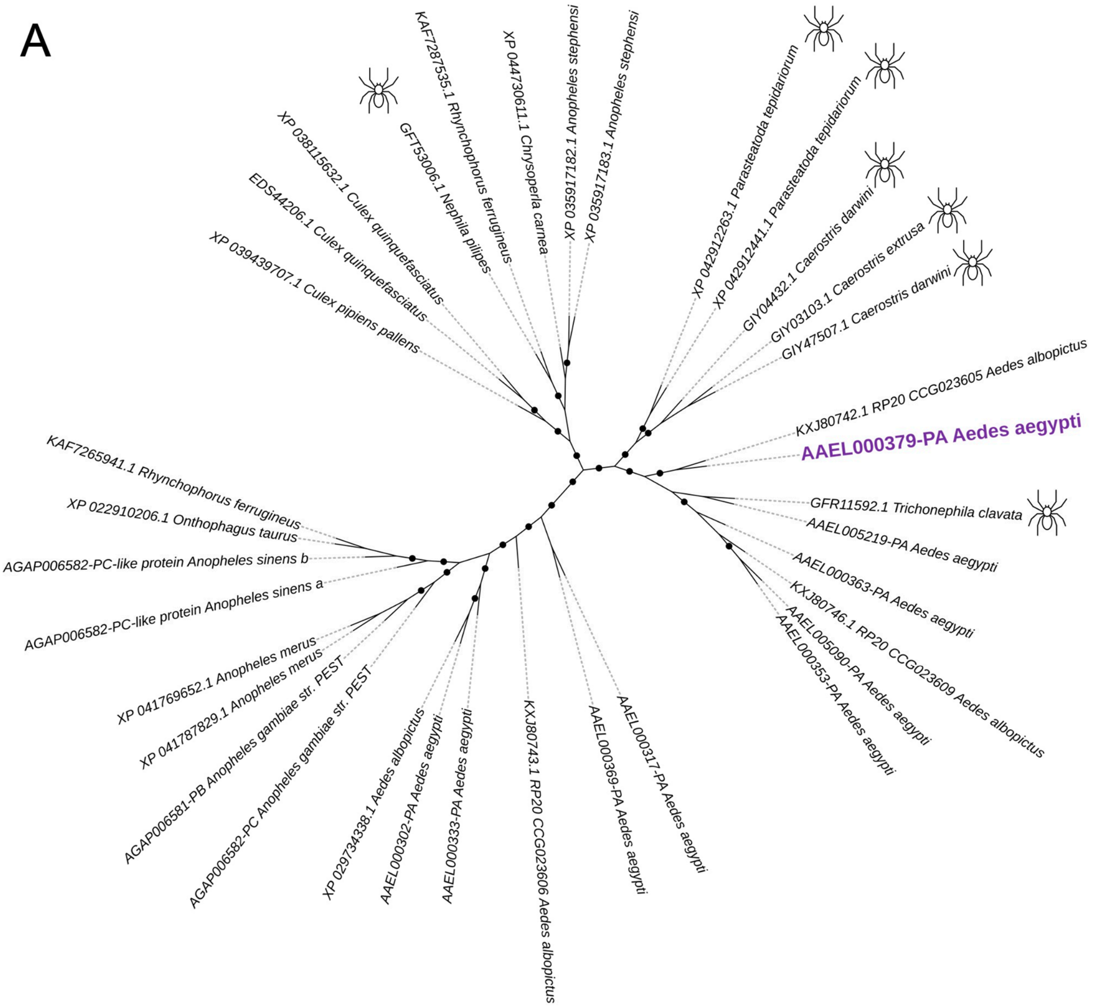

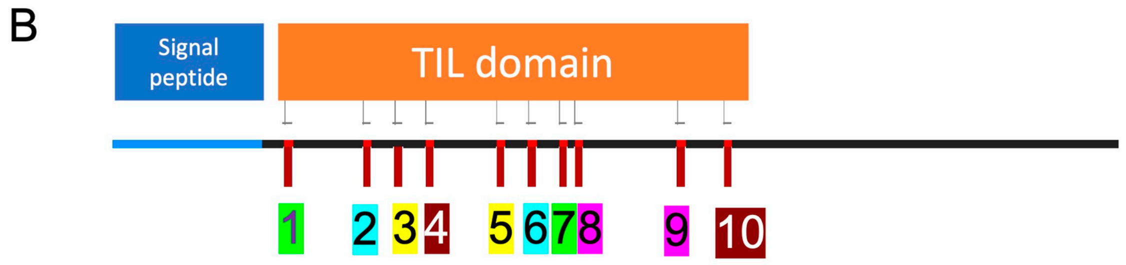

Supplementary Figure S1: (A) Unrooted phylogenetic tree of *Ae. aegypti* CRVP379 and closely related proteins obtained from the NCBI (nr database). CRVP379 is highlighted in bolded purple. Bootstrap values of 70% or higher are highlighted with dots. Proteins from spiders are highlighted with icons. (B) Schematic representation of key features from *Ae. aegypti* CRVP379. Cysteine residues predicted to form disulfide bridges are highlighted in the same color.

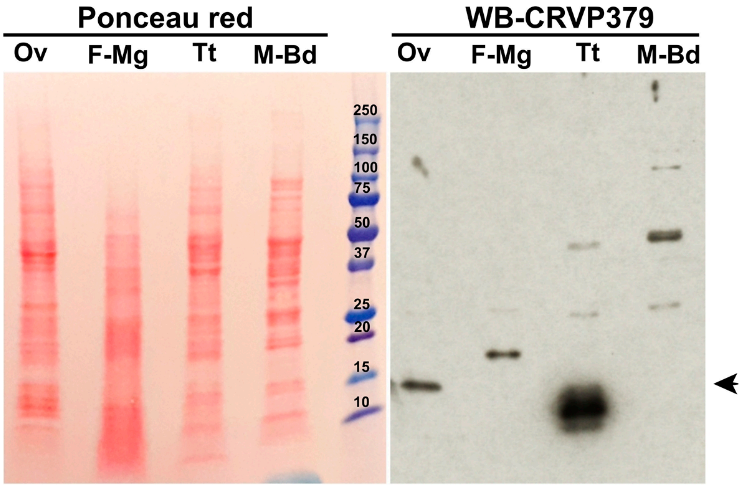

Supplementary Figure S2: Detection of CRVP379 with Western blotting utilizing anti-CRVP379 antibody. Ov: ovaries; F-mg: female midgut; T: male testes; M-bd: male body without testes. Left panel: Ponceau staining; right panel: Western blotting. Arrow indicates CRVP379 band.

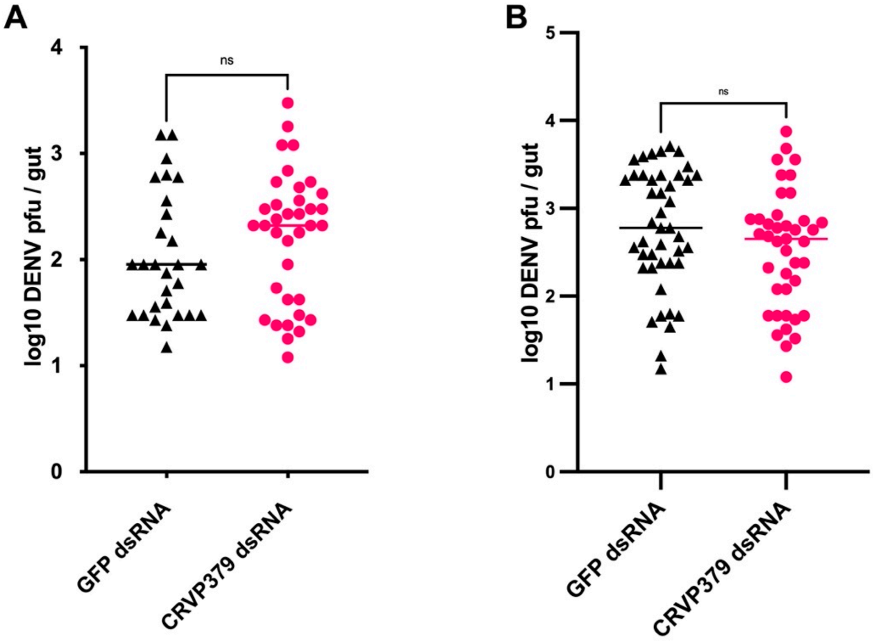

Supplementary Figure S3: Effect of CRVP379 gene silencing on DENV titers in the *Ae. aegypti* Rockefeller midgut. (A) 4 days after infection; (B) 7 days after infection. There was no statistically significant difference in DENV titers between control mosquitoes injected with GFP dsRNA and CRVP379 dsRNA (Mann Whitney test: p=0.2645 for day 4, p=0.1239 for day 7).

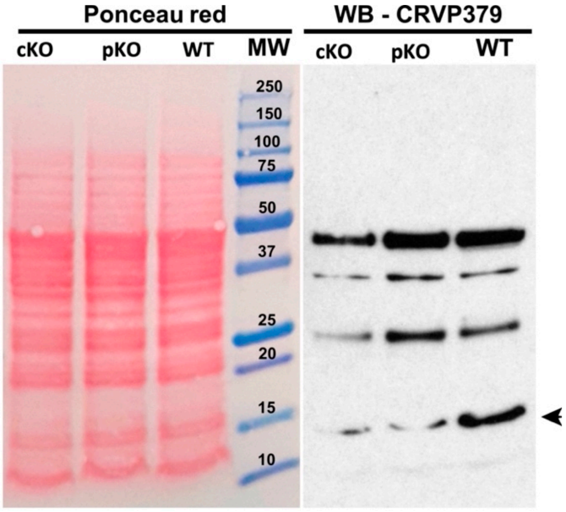

Supplementary Figure S4: Detection of CRVP379 with Western blotting utilizing anti-CRVP379 antibody in wild-type, CRVP-pKO, and CRVP-cKO whole bodies. Left panel: Ponceau staining, right panel: Western blotting. Arrow indicates CRVP379 band.

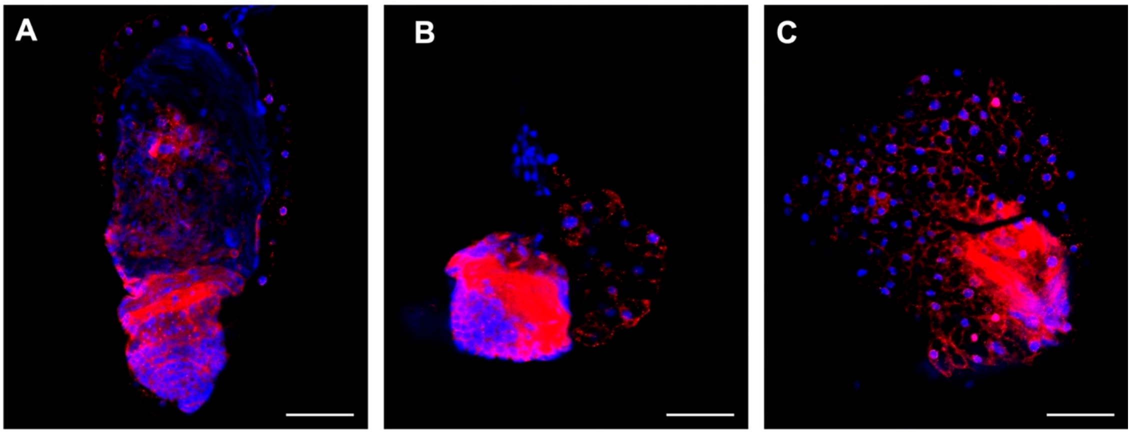

Supplementary Figure S5: Immunofluorescent detection of CRVP379 in the testes of *Ae. aegypti*. Anti-CRVP379 antibody: red; DAPI: blue. (A) *Ae. aegypti* Liverpool. (B) CRVP-pKO. (C) CRVP-cKO, 20X magnification, Bar scale = 100µm.

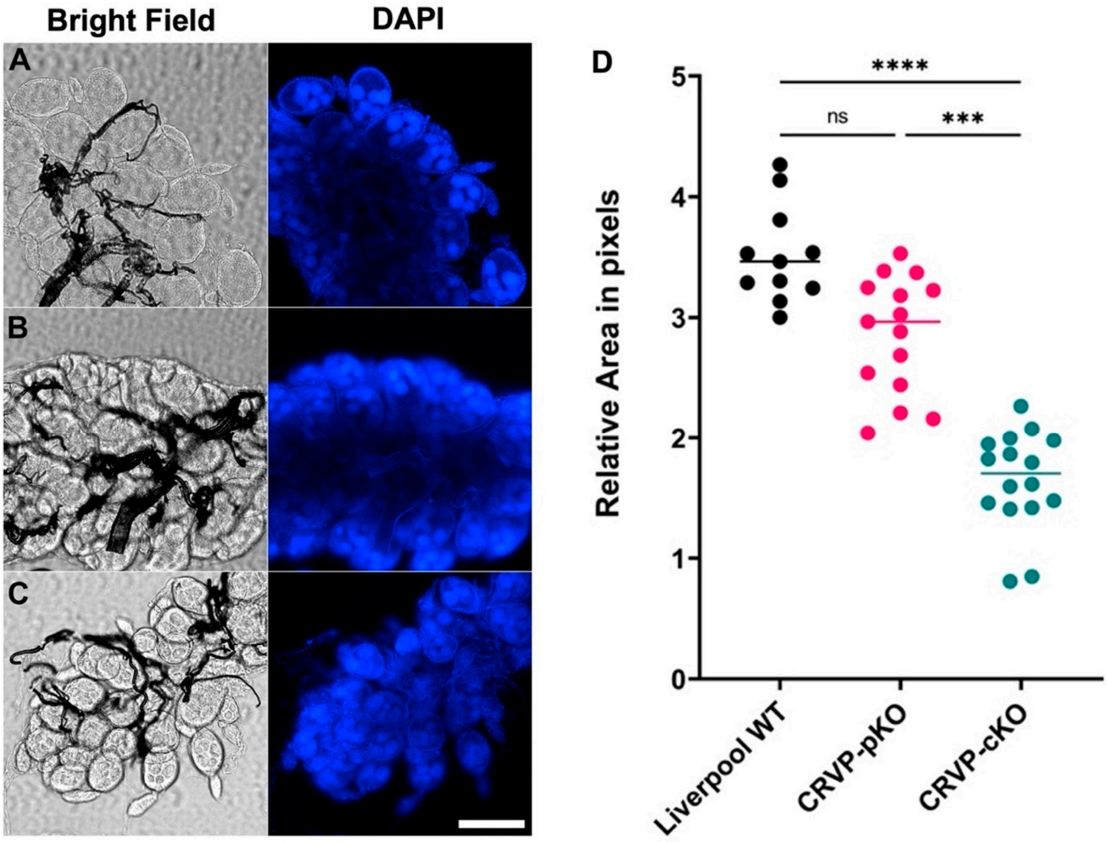

Supplementary Figure S6: (A) General morphology of follicles in wild-type (A) Liverpool, (B) CRVP-Pko (C) CRVP-cKO ovaries, 20X magnification (D) Follicle size as measured by the area (pixels) tool in ImageJ Fiji for wild-type Liverpool, each data set represents one follicle, CRVP-pKO, and CRVP-cKO ovaries (Dunn's multiple comparison test, **** $p<0.0001$ between Liverpool and CRVP-cKO, *** $p= 0.0005$ between CRVP-pKO and CRVP-cKO. ns- non-significant). Bar scale = 100 µm.

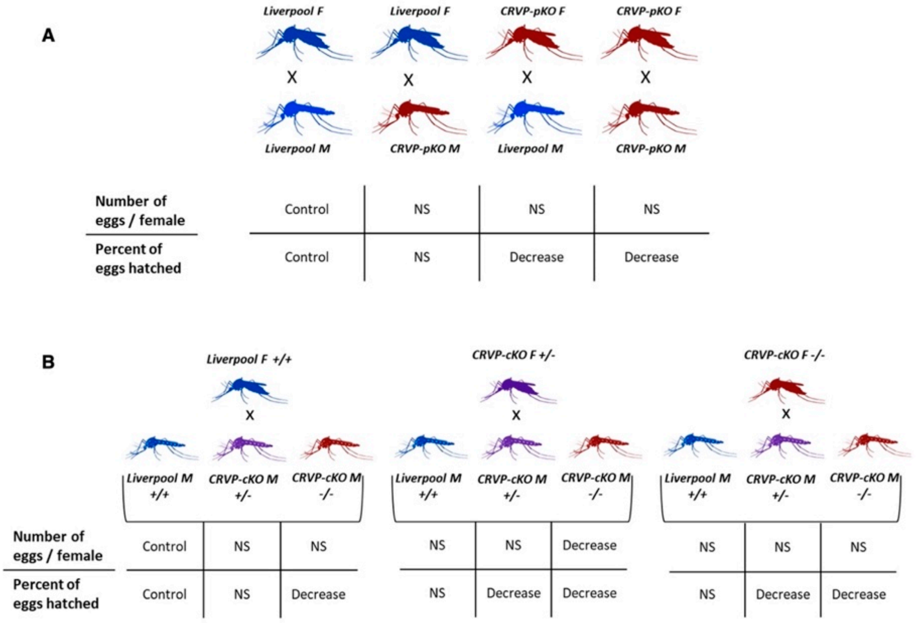

Supplementary Figure S7: Graphical representation of various CRVP379 crosses; F-female, M- male, NS-No significant change compared to WT Liverpool cross, Decrease- Statistically significant decrease A) CRVP-pKO crosses B) CRVP-cKO crosses; WT Liverpool +/+, CRVP379 heterozygous genotype +/-, CRVP379 homozygous genotype -/-. Image generated with Biorender, (Agreement number *AA240G3DTD, IX240G37A0)*

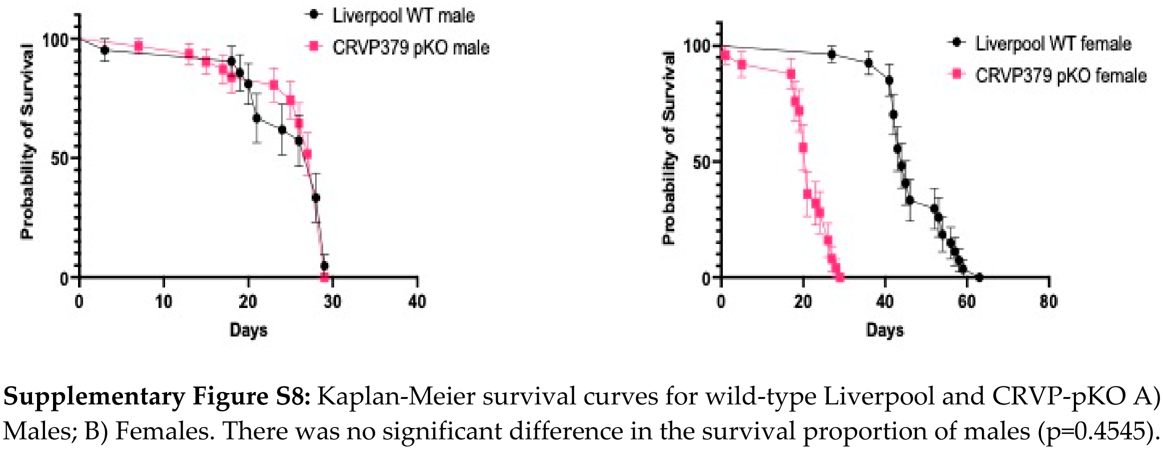

Supplementary Figure S8: Kaplan-Meier survival curves for wild-type Liverpool and CRVP-pKO A) Males; B) Females. There was no significant difference in the survival proportion of males (p=0.4545). CRVP-pKo females had a significantly shorter lifespan than did Liverpool females (p< 0.0001, log-rank Mantel-Cox test).

SUPPLEMENTARY FILE S1

Table S1 Protein sequences of CRVP379 from wild type and mutant lines

Primer/ Guide RNA	Sequence
CRVP_seq_F / CRVP_qpcr_F	5'-CGGCACCAAACAGTTCAAG-3'
CRVP_seq_R	5'-TCCTAGCCGAACAATGGCG-3'
CRVP_qpcr_R	5'-CGCCGTAACGCAAGTGTAG-3'
CRVP_RNAi_F	5'-<u>TAATACGACTCACTATAGG</u>GCGCTAATCTACACTTGCGTT-3'
CRVP_RNAi_F	5'- <u>TAATACGACTCACTATAGG</u>GAGCCACCACAATGGAC-3'
GFP_RNAi_R	<u>5'-TAATACGACTCACTATAGGG</u>TTCATCTGCACCACCGGC-3'
GFP_RNAI_F	5'<u>- TAATACGACTCACTATAGGG</u>CTGGTAGTGGTCGGCGAG-3'
Rps17_F	5'-GCAGACCACCATTGAACACA-3'
RPS17_R	5'- CACGTCCGGTCAGCTTCTTG-3'
gRNA 8	5'-AACGAGGTGTACCAGGAATGTGG-3'
gRNA 4	5'-CCAGTGCGTACCGAGTTCCAAGT-3'
gRNA 1	5'- CCTCCGCTGGTAATACCCGTACC-3'

>CRVP_WT

MKLLISLAVIALIYTCVTASNFCSGPNEVYQECGSACEKTCAGLGANQTCNEKCVPGCFCADGFVRLNHSGQCVPSSKCP
KVRVRRAPEPLPPLVIPVPIPIPIPVPPIVRPRGPLWWLRPPPPPLFG

>CRVP379-pKO

MKLLISLAVIALIYTCVTASNFCSGPNEVYQECGSACEKTCAGLGANQTCNEKCVPGCFCADGFVRLNHSGQCVPRPRG
PLWWLRPPPPPLFG

>CRVP_379-cKO

MKLLISLAVIALIYTCVTASNFCSGPNEVYQAKCGSACANVAQLARRHVPALESTSRATKSAFRDASARMGSFGSTIAAS
AYRVPSVPK
